# Supplementary material for: A pilot trial to evaluate the clinical usefulness of contrast-enhanced ultrasound in predicting renal outcomes in patients with acute kidney injury
Source: PLoS One. 2020 Jun 24;15(6):e0235130. doi: 10.1371/journal.pone.0235130 (PMC7313752; doi:10.1371/journal.pone.0235130)
Supplement: S1 Table — (DOCX) [file pone.0235130.s001.docx]

### S1 Table. Correlations between TIC parameters and FENa or lowest urine output

|  | FENa | | Lowest urine output | |
| --- | --- | --- | --- | --- |
|  | *r* | *P-value* | *r* | *P-value* |
| TIC parameters |  |  |  |  |
| Cortex |  |  |  |  |
| WIS (dB/sec) | -0.248 | 0.122 | -0.075 | 0.645 |
| TTP (s) | 0.067 | 0.680 | 0.163 | 0.315 |
| PI (dB) | -0.010 | 0.950 | -0.166 | 0.306 |
| AUC (dB) | 0.053 | 0.746 | -0.240 | 0.135 |
| MTT (s) | 0.162 | 0.319 | -0.202 | 0.212 |
| FWHM (s) | 0.135 | 0.406 | -0.227 | 0.159 |
| RT (s) | 0.170 | 0.293 | -0.138 | 0.397 |
| Medulla |  |  |  |  |
| WIS (dB/sec) | 0.032 | 0.845 | -0.155 | 0.340 |
| TTP (s) | -0.065 | 0.690 | 0.079 | 0.630 |
| PI (dB) | -0.045 | 0.784 | -0.147 | 0.364 |
| AUC (dB) | -0.088 | 0.587 | -0.143 | 0.380 |
| MTT (s) | 0.020 | 0.900 | -0.102 | 0.529 |
| FWHM (s) | 0.056 | 0.732 | -0.077 | 0.637 |
| RT (s) | -0.094 | 0.564 | -0.124 | 0.447 |

TIC = time-intensity curve, OR = odds ratio, CI = confidence intervals, WIS = wash in slope, TTP = time to peak intensity, PI = peak intensity, AUC = area under the time-intensity curve, MTT = mean transit time, FWHM = time for full width half max, RT = rise time.
