# Supplementary material for: A pilot trial to evaluate the clinical usefulness of contrast-enhanced ultrasound in predicting renal outcomes in patients with acute kidney injury
Source: PLoS One. 2020 Jun 24;15(6):e0235130. doi: 10.1371/journal.pone.0235130 (PMC7313752; doi:10.1371/journal.pone.0235130)
Supplement: S2 Table — (DOCX) [file pone.0235130.s002.docx]

### S2 Table. Comparison of TIC parameters between patients with intrinsic AKI and patients with prerenal or postrenal AKI

|  | IntrinsicAKI (n = 34) | Prerenal or postrenal AKI (n = 14) |  |
| --- | --- | --- | --- |
|  |  |  |  |
| TIC parameters |  |  | *P-value* |
| Cortex |  |  |  |
| WIS (dB/sec) | 0.88 (0.62) | 0.82 (0.22) | 0.711 |
| TTP (s) | 42.74 (10.63) | 44.07 (12.66) | 0.712 |
| PI (dB) | 17.76 (3.16) | 17.41 (3.03) | 0.729 |
| AUC (dB) | 2179 (465.62) | 2112 (583.83) | 0.680 |
| MTT (s) | 72.65 (16.13) | 68.88 (15.36) | 0.459 |
| FWHM (s) | 116.18 (22.00) | 111.49 (25.72) | 0.526 |
| RT (s) | 17.12 (4.96) | 16.50 (3.22) | 0.669 |
| Medulla |  |  |  |
| WIS (dB/sec) | 0.86±0.68 | 0.99±0.87 | 0.585 |
| TTP (s) | 44.65 (11.00) | 46.11 (14.39) | 0.705 |
| PI (dB) | 17.82 (3.48) | 17.77 (3.51) | 0.967 |
| AUC (dB) | 2246 (510.98) | 2300 (516.27) | 0.738 |
| MTT (s) | 75.45 (13.82) | 74.68 (13.03) | 0.861 |
| FWHM (s) | 120.08 (22.12) | 118.38 (27.52) | 0.822 |
| RT (s) | 18.18 (5.44) | 17.87 (3.92) | 0.848 |

Values are mean with standard deviation in parentheses. TIC = time-intensity curve, OR = odds ratio, CI = confidence intervals, WIS = wash in slope, TTP = time to peak intensity, PI = peak intensity, AUC = area under the time-intensity curve, MTT = mean transit time, FWHM = time for full width half max, RT = rise time.
