# Supplementary material for: A pilot trial to evaluate the clinical usefulness of contrast-enhanced ultrasound in predicting renal outcomes in patients with acute kidney injury
Source: PLoS One. 2020 Jun 24;15(6):e0235130. doi: 10.1371/journal.pone.0235130 (PMC7313752; doi:10.1371/journal.pone.0235130)
Supplement: S3 Table — (DOCX) [file pone.0235130.s003.docx]

**S3 Table. Difference of TIC parameters according to the presence of underlying CKD**

| TIC parameters | Underlying CKD  (n = 23) | No underlying CKD (n = 25) | *P*-value |
| --- | --- | --- | --- |
| Cortex |  |  |  |
| WIS (dB/sec) | 1.09 (1.43) | 0.78 (0.19) | 0.89 |
| TTP (s) | 42.94 (12.78) | 43.30 (9.66) | 0.91 |
| PI (dB) | 16.50 (3.25) | 18.72 (2.58) | 0.01 |
| AUC (dB) | 1886 (509.7) | 2411 (330.2) | <0.001 |
| MTT (s) | 65.52 (17.95) | 77.10 (11.37) | 0.02 |
| FWHM (s) | 104.10 (28.40) | 125.00 (17.39) | 0.01 |
| RT (s) | 16.12 (5.29) | 17.70 (3.57) | 0.23 |
| Medulla |  |  |  |
| WIS (dB/sec) | 1.08 (1.03) | 0.72 (0.16) | 0.52 |
| TTP (s) | 43.74 (13.12) | 46.32 (10.88) | 0.24 |
| PI (dB) | 16.65 (3.52) | 18.87 (3.07) | 0.02 |
| AUC (dB) | 1991 (497.40) | 2511 (463.10) | <0.001 |
| MTT (s) | 70.26 (15.34) | 79.80 (9.88) | 0.02 |
| FWHM (s) | 108.9 (25.81) | 129.4 (16.25) | 0.004 |
| RT (s) | 16.95 (4.85) | 19.13 (5.01) | 0.14 |

Values are mean with standard deviation in parentheses. TIC = time-intensity curve, CKD = chronic kidney disease, WIS = wash in slope, TTP = time to peak intensity, PI = peak intensity, AUC = area under the time-intensity curve, MTT = mean transit time, FWHM = time for full width half max, RT = rise time.
