# Supplementary material for: A pilot trial to evaluate the clinical usefulness of contrast-enhanced ultrasound in predicting renal outcomes in patients with acute kidney injury
Source: PLoS One. 2020 Jun 24;15(6):e0235130. doi: 10.1371/journal.pone.0235130 (PMC7313752; doi:10.1371/journal.pone.0235130)
Supplement: Attachment — Submitted filename: Review, Arp 6th.docx [file pone.0235130.s004.docx]

Dear Prof. Tatsuo Shimosawa,

Academic Editor,

PLOS One

The manuscript of “A pilot trial to evaluate the clinical usefulness of contrast-enhanced ultrasound in predicting renal outcomes in patients with acute kidney injury” is interesting to develop and establish renal vessel CEUS. I expect the new method is important for evaluation of real-time renal function of AKI as well as CKD.

I have several questions about methods.

Comments;

1) There are a lot of unique abbreviations. I recommend to make abbreviation section.
2) I recommend to give a figure of the examination protocol. It is useful to understand the examination and the result data.
3) How many sonographers did exam the protocol?
4) It was written “the transducer was manually held in the same scanning plane while patients were instructed to perform only shallow breathing to minimize the variation caused by motion” in Materials and Methods. I think each holding time was very long because the protocol time should include baseline, bolus shot and follow up. And I concern the base line data should get much different when the angle of the transducer moves just a little. How they contrive ways to keep the transducer in the same place and the same angle?
