# Supplementary material for: A pilot trial to evaluate the clinical usefulness of contrast-enhanced ultrasound in predicting renal outcomes in patients with acute kidney injury
Source: PLoS One. 2020 Jun 24;15(6):e0235130. doi: 10.1371/journal.pone.0235130 (PMC7313752; doi:10.1371/journal.pone.0235130)
Supplement: Attachment — Submitted filename: Response_to_editor_and_reviwer_s_commonets.docx [file pone.0235130.s005.docx]

To the editor

We would like to thank the editor and reviewers for their insightful comments. Their comments definitely helped to improve the quality of our manuscript. We have tried our very best to answer the reviewers’ specific points. The reviewer's comments are in blue and our answers are in black. We hope our revisions improve the paper such that it is worthy of publication in PLOS ONE. Thank you.

**Point by point response to reviewers’ comments**

**[1] Journal requirements:**

When submitting your revision, we need you to address these additional requirements. Please ensure that your manuscript meets PLOS ONE's style requirements, including those for file naming.

In response to this comment, we made sure that our manuscript was correctly formatted, according to the PLOS ONE's style requirements.

**[2] Reviewer #1 comments:**

1. Since CEUS is not a widely used technology and even when it is used, the methodology varies widely, I recommend a brief description of different parameters and what each of them indicate before proceeding to further discussion of methodology. For example, what does WIS or MTT after an injection of a bolus of contrast media indicate and what do we expect to see in various forms of AKI.

We thank the reviewer for this helpful comment. However, there is limit to the number of characters in the text and how the CEUS parameters appear in various forms of AKI is unknown yet. We can only speculate what these parameters mean in clinical settings. To avoid redundancy, we additionally described the meanings and clinical implications of the quantitative CEUS parameters in the discussion section.

2. Estimated GFR by CKD Epi formula should only be used to determine the baseline renal function and not after AKI.

Thank you for the comment. We deleted the “eGFR at AKI occurrence” and the “lowest eGFR” from Table 1. Instead we included the “serum creatinine at AKI occurrence” and the “highest serum creatinine” in Table 1.

3. It would be helpful to know the cause of AKI and the urine output in these patients. Data on the number of patients requiring RRT and a scale of severity of illness would also be valuable. I am surprised that mortality was zero.

We added the causes of AKI (prerenal, intrinsic, and postrenal) in Table 1 and also described it in more detail in the result section. The lowest urine output was added in Table 1. The AKI severity was shown as the KDIGO AKI stage in Table 1 and was described in the result section as “Among them, 25 (52%) patients were diagnosed as KDIGO stage 3 AKI, 11 (23%) patients received RRT, 13 (27%) patients achieved functional recovery of AKI, and 18 (38%) patients showed CKD progression. Ten patients (20.8%) had urine output less than 500 mL per day, two patients (4.2%) developed anuria during the course of AKI, and the in-hospital mortality rate was 0%.”

As the reviewer commented, the mortality rate was zero. This was because it was difficult to enroll patients in severe illness, since patients who are hemodynamically unstable and are in a clinically downhill course cannot undergo CEUS. This was described in the limitation.

4. Do CEUS variables differentiate AKI causes or correlate with FENa or FEUrea or response to volume expansion?

As the reviewer suggested, we analyzed the correlation coefficient between the CEUS variables and FENa or urine output. None of the CEUS variables showed statistically significant correlation coefficients (supplement table 1). The data on FEUrea or response to volume expansion was not collected in this study.

The CEUS variables were compared between patients with intrinsic causes and those with prerenal or postrenal causes. None of the TIC parameters showed statistically significant difference between patients with intrinsic AKI and patients with prerenal or postrenal AKI (Supplement table 2). Therefore the CEUS variables did not differentiate AKI causes.

The description for supplement tables 1 and 2 were added in the result section.

5. I am not sure if logistic regression is the right test, considering CEUS parameters are not completely independent of one another. If you were including one CEUS variable in the model and were adding other potential predictors of AKI outcome, that might have been useful.

The statistical analysis in our study was performed by statistician in our university. We consulted with the statistician about performing the multivariate analysis, and received this answer as follows.

“In statistics, the ‘one in ten rule’ is a rule of thumb for how many predictor parameters can be estimated from data when doing regression analysis, while keeping the risk of overfitting low. The rule states that one predictive variable can be studied for every ten events. In this study, the outcome events were need of RRT, AKI recovery, and CKD progression. The number of the events was small, which was all less than 20. Therefore, to keep the overfitting low and make the result reliable, only one predictor can be included in the logistic regression analysis. That is why we only performed a univariate logistic regression analysis”.

We agree with the reviewer’s comment that a multivariate analysis is needed to demonstrate the predictive value of CEUS parameters. But as the number of patients and number of outcome events were small, we could not perform a multivariate analysis. We added this as a limitation in the discussion section.

“…As the numbers of patients and outcome events were small, we could not perform a multivariate logistic analysis. Therefore the predictability of the CEUS perfusion parameters cannot be generalized yet…..”

6. Considering medulla receives only about 15% of renal blood flow and has very slow velocities and transition times, I am very surprised to see comparable numbers for cortex and medulla. The time to peak is almost identical for the cortex and medulla.

Currently, there is no CEUS study for medullary perfusion in human. A recent animal study showed that medullary PI and AUC were about one-third of that of cortex in healthy dogs, on the other hand, medullary PI and AUC were almost the same as cortex values in dogs with AKI (J Small Anim Pract. 2019 Aug;60(8):471-476). The increase in medullary PI and AUC seen in dogs with AKI, which suggests increased medullary perfusion, is similar to our results. We speculate that a medullary blood congestion and consequent slowing of the blood flow occurred in our patients with AKI. This medullary congestion was shown to be a key event in ischemic AKI murine model (Kidney Int. 1984 Sep;26(3):283-93).

7. In the image provided the ROI are much smaller than what we use and other investigators have used. I am wondering why larger ROI are not used, at least for the cortex.

According to the referenced literature (Current Drug Targets 2009;10(12):1184-9), the pattern and quantity of blood flow and tissue blood volume in different regions within the organ, i.e., cortex separately from medulla, can be determined within a few minutes with CEUS, which is particularly desirable in clinical situations in which patient transfer out of the intensive care unit is an issue. By providing information on regional changes in renal blood flow, CEUS has the potential to serve as a helpful tool in the diagnostic work up of patients with AKI.

We wanted to find out if there is a perfusion difference between cortex and medulla or to look at corticomedullary perfusion ratios of CEUS parameters. Therefore, we thought that the ROI of the cortex and medulla should be drawn in the same size and shape as possible. However, the medulla, unlike a cortex, is not easy to draw an entire ROI. So, we analyzed by drawing three ROIs of the same size in the cortex and medulla in the upper, middle, and lower regions. In order to include only the microvasculature in the outer cortex according to very strict criteria, many reported data obtained the ROI of the cortex in the same way as in our study.

8. The image intensity needs to be standardized for the renal artery, aorta or left ventricle intensity. That would eliminate the issues with errors in volume of contrast, extravasation from the vein, etc.

We agree with to the reviewer's comment, in that a suitable arterial input function is usually not available in the bolus-administration mode. In addition, some perfusion parameters are severely influenced by ultrasound attenuation in intervening tissue. However, the heart, renal parenchyma and bowel wall represent the most established applications of CEUS in the quantification of organ perfusion in humans. Until recently, the quantitative analysis of renal tissue perfusion, measured by CEUS, has been performed without quantification standardization. In addition, although manual injection might potentially predispose to lower accuracy in contrast infusion, the majority of studies used manual injection. Therefore, we did not standardize the image intensity of the renal parenchyme to renal artery, aorta or left ventricle intensity.

9. None of the parameters from CEUS have good AUC or reasonable sensitivity or specificities.

In our study, we found that the AUC in MTT, WIS, PI values were 0.75, 0.72, and 0.69, respectively. Although diagnostic accuracy is not satisfactory, our preliminary findings are encouraging for the performance of quantitative CEUS analysis as a screening test for patients with AKI. Many factors can affect CEUS quantitative analysis, including the frequency, the mechanical index, the analytical software, the contrast dose, patient’s factor, the injection velocity of contrast agents and so on. In this regard, further standardization and investigation are needed.

10. Under discussion, I recommend more details on interpretation of results. In other words, it would be helpful to know why higher or lower values of certain parameters indicate a more severe degree of tissue injury. What is the clinical implication of these findings?

We thank the reviewer for this helpful comment. In response to this comment, we added the following sentence in discussion:

“Our study found that the MTT at the renal cortex was increased in patients requiring RRT than that of patients not requiring RRT. Considering that MTT indicates the average time taken by blood to pass through the capillary network, this finding indicated that, less contrast microbubbles entered the renal cortex microvascular bed with slow perfusion in unit time in patients requiring RRT compared with those not requiring RRT. Harrois *et al* showed that the greater the alteration in MTT is, the higher is the risk of severe AKI, indicating that MTT seems to be mostly linked to intrarenal hemodynamics (Crit care 2018;22:161). PI reflects the quantity of contrast agent microbubbles in the vascular bed of the organ, while WIS reflects the early quantity and velocity during contrast agent perfusion. These two parameters are associated with the degree of vascularization. The WIS at the renal cortex and the PI at the renal medulla was higher in the AKI recovery group than that in the non-recovery group. This finding suggests that better vascularization at the renal cortex and medulla is associated with AKI recovery. In addition, lower PI at the renal medulla was associated with CKD progression, which meant that reduced medullary perfusion is not protective against tissue recovery. This finding is consistent with a previous report which showed that PI at the renal medulla decreased as the CKD stages progressed (Int Heart J 2010;51:176-82). Increased RT indicates delay in rise in echogenicity, which is related with increased resistance of glomeruli and peritubular capillaries (Int Heart J 2010;51:176-82). In this study, increased RT at the cortex was associated with severe AKI stage and need of RRT. This finding appears to reflect the delayed cortical perfusion of patients with severe AKI. The AUC means the total volume of blood flow. In our study, the increase in AUC at renal medulla, which indicates medullary blood congestion, was associated with CKD progression. It is difficult to analyze the clinical implication of this finding, since the AUC is dependent on the capillary resistance, retention time and total capillary volume. We can only speculate that an alteration in the medullary capillary density may be associated with progressive renal injury. However, since the sensitivity, specificity, and reliability of medullary PI, AUC, and RT were low, the clinical significance of these parameters needs caution in interpretation.”

**[3] Reviewer #2 comments:**

1. There are a lot of unique abbreviations. I recommend to make abbreviation section.

As the reviewer commented, we added an abbreviation section in the end of the abstract.

2. I recommend to give a figure of the examination protocol. It is useful to understand the examination and the result data.

In response to this comment, we made a figure of the examination protocol and added the sentence in the material and methods:

“The examination protocol is illustrated in Figure 2.”

3. How many sonographers did exam the protocol?

All CEUS examinations were performed by one experienced radiologist to ensure the stability of measurements.

This sentence has already been included in the Materials and Methods.

4. It was written “the transducer was manually held in the same scanning plane while patients were instructed to perform only shallow breathing to minimize the variation caused by motion” in Materials and Methods. I think each holding time was very long because the protocol time should include baseline, bolus shot and follow up. And I concern the base line data should get much different when the angle of the transducer moves just a little. How they contrive ways to keep the transducer in the same place and the same angle?

Examinations were performed as followed: First, conventional B-mode US was performed to scan the kidney and to observe the size, position and echogenicity. During B-mode scanning, maximal longitudinal scanning was chosen as the ideal plane for CEUS. Second, the US system was then switched to contrast mode for CEUS examination. Contrast agent was injected manually as a bolus over 2 seconds followed by a flush of 10 mL saline solution. The entire CEUS process was recorded for each patient from the time of injection, until no apparent agent was observed. Our study analyzed images of kidney perfusion after the contrast agent was given, focusing only on cortex and medulla perfusion. Conventional ultrasound examination is intended to confirm the approximate target organ location for contrast-enhanced ultrasound examination to be performed later, and there is no special baseline data. In addition, the kidney is an organ located in the retroperitoneal space, and unlike other organs in the abdominal cavity, there is less movement caused by patient's breathing. In addition, we specifically instructed the patients regarding their respiration control before the CEUS examination was performed. We found that our study showed a relatively stable perfusion graph except for patients with severe respiratory disease.

5. There is not section of COI nor funding.

We entered a financial disclosure statement and COI statement during the submission process.

**[4] Reviewer #3 comments:**

Thank you for your informative comments.
