# Supplementary material for: A pilot trial to evaluate the clinical usefulness of contrast-enhanced ultrasound in predicting renal outcomes in patients with acute kidney injury
Source: PLoS One. 2020 Jun 24;15(6):e0235130. doi: 10.1371/journal.pone.0235130 (PMC7313752; doi:10.1371/journal.pone.0235130)
Supplement: Attachment — Submitted filename: Response-revision 2.doc [file pone.0235130.s006.doc]

To the editor

We would like to thank the editor and reviewers for their insightful comments. Their comments definitely helped to improve the quality of our manuscript. We have tried our very best to answer the reviewers’ specific points. The reviewer's comments are in back and our answers are in blue. We hope our revisions improve the paper such that it is worthy of publication in PLOS ONE. Thank you.

**Point by point response to reviewers’ comments**

**[1] Reviewer #1 comments:**

Reviewer #1: Dear Editor,

I reviewed the revised manuscript and found the changes very helpful. Here are my remaining concerns and suggestions for improvement,

1. I am not satisfied with the response provided regarding the use of logistic regression. The independent variables (predictors) in logistic regression should have minimal or no collinearity. They need to be independent of one another and not to correlate with each other. Therefore, different CEUS parameters cannot be used in the same logistic regression analysis as they are not independent of one another.
■ We agree with the reviewer’s comment that CEUS parameters are not independent of each other. The logistic regression analysis was univariate, and included “only one” CEUS parameter for each outcome. Thus, multiple CEUS parameters were not included in a same analysis.

As the reviewer pointed out in the 1^st^ review, adding other clinical variables would be useful to clearly demonstrate the association between CEUS parameters and outcomes. But as the number of patients and number of outcome events were small, we could not perform a multivariate analysis.

As our description for the statistical analysis and the results on the logistic regression may confuse the reader, we tried to clarify this by adding the term “univariate” and a sentence “Since CEUS parameters are not completely independent of one another, we included one CEUS parameter for each outcome in the logistic regression analysis.”

2. I believe a dot plot of actual values of selected CEUS parameters (those that were significantly associated with the study outcomes) for different AKI stages or for those who required RRT versus those who didn't would be necessary. A visual representation of the differences and overlaps between the groups would be more useful than OR.
■ We thank the reviewer for this helpful comment. We made a dot plot of actual values of selected CEUS parameters as Figure 4.

3. CEUS studies were probably performed during different stages of AKI evolution. While a patient may have had their study on day one with minimal rise in serum Cr, another patient may have had it at the peak and a third one while recovering. Reporting the mean difference between the time of CEUS and time of peak serum Cr or nadir of urine output would be helpful in confirming the utility of this tool in prognostication of AKI.
■ We agree with the reviewer’s comment. We added the mean, standard deviation, and range of the time of CEUS and time of peak serum Cr in the results, and added this as a limitation of this study.

“The duration between the time of CEUS and the time of peak serum creatinine was 3.8 ± 3.9 days (range, 0 – 14 days).”

4. The imaging study of one kidney occurred immediately after injection of the contrast bolus, while the second kidney was images later on. Certain parameters, such as time to peak cannot be obtained for the second kidney. Additionally, since contrast was given as a bolus and not a continuous infusion, it is expected to have lower image intensity during the study of the second kidney. Please clarify if the data used is only from the first kidney or both and if there were differences between the two.
■ The contrast agent was injected before examination of each kidney. The right kidney was examined first and, after about 20 min, the same CEUS examinations (including injection of SonoVue) were performed for the left kidney. We added this to clarify how the CEUS was performed.

“The contrast agent was injected before examination of each kidney. The right kidney was examined first and, after about 20 min, the same CEUS examinations (including injection of SonoVue) were performed for the left kidney.”

We obtained CEUS parameters for both kidneys respectively and additionally calculated the average value of each parameter from both kidneys of each individual. Because the amount of data is too large and difficult to express, only the average value was shown in the paper. A few previous studies conducted in the same way by Wang L et al. [Biomed Res Int. 2015 and J Nephrol 2015]. Although not shown in the paper, we can see that there is no significant difference in the CEUS values of the right and left kidneys, and similar results have been confirmed in some recently published papers [Clin Hemorheol Microcirc 2016;62:229-38, Br J Radiol 2014 Oct;87(1042):20140350. doi: 10.1259/bjr.20140350]. And it has been reported that contrast agent concentrations and GFR are not significantly different between the left and the right kidney [J Vet Med Sci 2016 Feb;78:239-44].

In accordance with this comment, we have added the following sentence to clarify the meaning.

“The final reported results of CEUS parameters represent the average value of each parameter from both kidneys of each individual.”

**Minor changes**

Under Study Design and Patients,
1. Line 84, Adult patients?

■ Yes, only adult patients were enrolled. We added the exclusion criteria for age.

2. Line 84, Change our hospital to the actual name of the hospital

■ We added the actual name of the hospital.

3. Line 96, move number of patients to results.

■ We moved the number of patients to the result section.

4. Line 98, specify what was measured in urine.

■ Urine sodium, creatinine, and protein levels were measured. We specified this as the reviewer commented.

5. Line 100. Change renal function to "baseline renal function", since you won't be using the equation for measuring GFR after AKI.

■ We revised the term as “baseline renal function”.

6. Line 140-105, what was the frequency of follow up?

■ The frequency of follow-up differed between patients. If the patient was cared in the outpatient clinic, the follow-up interval was determined according to the physician’s decision, and ranged from 2 days to 1 week. If the patient was admitted in the hospital, the patient’s status was checked every day. If the patient’s renal function improved to his or her baseline function and the patient did not have CKD, the follow-up was stopped at 3 months post-AKI. Patients with CKD or non-recovery of AKI were followed-up more than 3 months post-AKI, and the follow-up interval was determined according to the physician’s decision.

This was briefly described in the method section.

7. Line 106, move AKI staging to where the definition is discussed.

■ We revised the sentence as the reviewer commented.

8. Under methods please indicate how the cause of AKI was determined.

■ The clinical context, history taking, physical examination, and interpretation of blood and urine laboratory findings were used for the differential diagnosis of the cause of AKI.

This was added in the method section.

9. Urine output is used in KDIGO definition of AKI. If that data was recorded and used in categorizing AKI stages, please indicate it under the methods section.

■ Not all patients were admitted to the hospital at the diagnosis of AKI. The urine output was calculated for admitted patients. Therefore the serum creatinine criteria was used for the definition and staging for AKI. We clarified such definition in the method section.

10. Table 1. I suggest moving CEUS data to a separate table under results.
■ We made a separate table for the CEUS data as the reviewer suggested.

**[2] Reviewer #2 comments:**

Reviewer #2: The paper is corrected better.
But I still have a question to ought to be clear, because the renal CEUS is not widely accepted yet. As all sonographic experiences were endured by one radiologist and manual probe supporting, how are they secure the standardization of the examination results?
■ After the injection of the contrast agent, all images and video clips were stored digitally on a hard disk system, and then transferred to a personal computer for further quantitative analyses using advanced US quantification software. As the CEUS parameters are obtained from a time-intensity curve, these parameters are calculated automatically by the software program. Therefore, we can expect that simply measuring these parameters will demonstrate a good agreement among observers, because it is different from characterizing a renal mass.

In general, it can be assumed that renal mass characterization will have greater interobserver variation than simple measurements of parenchymal perfusion. Recently the biggest study cohort of patients with unclear renal mass that were evaluated using CEUS showed a great interobserver agreement between two independent readers [Eur Radiol 2018;28:4542-49]. These findings are in line with several previous studies conducted about this topic.

Many CEUS studies about renal parenchymal perfusion were performed by a single radiologist [Transplant Proc 2009;41:3024-7, Clin Hemorheol Microcirc 2016;62:229-38, Antioxid Redox Signal 2017;27:1397-1411, Abdom Radiol 2018;43:1423-1431]. As previous studies did, our study was also conducted by one researcher.

**[3] Reviewer #3 comments:**

 ■ Thank you for reviewing our manuscript.
