# Supplementary material for: A pilot trial to evaluate the clinical usefulness of contrast-enhanced ultrasound in predicting renal outcomes in patients with acute kidney injury
Source: PLoS One. 2020 Jun 24;15(6):e0235130. doi: 10.1371/journal.pone.0235130 (PMC7313752; doi:10.1371/journal.pone.0235130)
Supplement: Attachment — Submitted filename: Response_to_reviwer_s_commonets 3.docx [file pone.0235130.s007.docx]

To the editor

We would like to thank the editor and reviewers for their insightful comments. Their comments definitely helped to improve the quality of our manuscript. We have tried our very best to answer the reviewers’ specific points. The reviewer's comments are in blue and our answers are in black. We hope our revisions improve the paper such that it is worthy of publication in PLOS ONE. Thank you.

**Point by point response to reviewers’ comments**

**[1] Reviewer #1 comments:**

Thank you for making all the suggested changes. The manuscript reads very well. I just wanted to mention that in my experience, the CEUS data for medulla differ significantly from that of cortex and I was surprised to see them identical in your study.

We thank the reviewer for this informative comment and agree with you. Unlike medulla, which normally receives less flow than cortex, we speculate that a medullary blood congestion and consequent slowing of the blood flow occurred in our patients with AKI. This medullary congestion is a hallmark of ischemic AKI in both experimental models (Kidney Int 1990; 37: 1240-1247, Kidney Int 1990; 38: 432-439, Kidney Int 1991; 40: 625-631, Lab Invest 1991; 65: 566-576) and in specimens obtained from biopsy or at autopsy (Kidney Int 1984; 26: 283-293). Currently, there is no CEUS study for medullary perfusion in humans with AKI. A recent animal study showed that medullary PI and AUC were about one-third of that of cortex in healthy dogs, on the other hand, medullary PI and AUC were almost the same as cortex values in dogs with AKI (J Small Anim Pract. 2019 Aug;60(8):471-476). The increase in medullary PI and AUC seen in dogs with AKI, which suggests increased medullary congestion, is similar to our results. However, our study had patients with mild AKI, whereas few papers on human CEUS had severe illness. Further studies with a larger sample size are needed for validation.

We added the following sentence about the medullary CEUS findings in discussion:

“In this study, the CEUS-driven TIC parameters of the medulla were not reduced compared to those of cortex. This was unexpected, as the renal medulla receives lower blood flow than the cortex (Seminars in Nephrology, Vol 39, No 6, November 2019, 520−529). We speculate that a medullary blood congestion and consequent slowing of the blood flow occurred in our patients with AKI. This medullary congestion is a hallmark of ischemic AKI in both experimental models (Kidney Int 1990; 37: 1240-1247, Kidney Int 1990; 38: 432-439, Kidney Int 1991; 40: 625-631, Lab Invest 1991; 65: 566-576) and in specimens obtained from biopsy or at autopsy (Kidney Int 1984; 26: 283-293). Currently, there is no CEUS study for medullary perfusion in humans with AKI. A recent animal study showed that medullary PI and AUC were about one-third of that of cortex in healthy dogs, on the other hand, medullary PI and AUC were almost the same as cortex values in dogs with AKI (J Small Anim Pract. 2019 Aug;60(8):471-476). The increase in medullary PI and AUC seen in dogs with AKI, which suggests increased medullary congestion, is similar to our results. Further studies with a larger sample size are needed for validation of medullary CEUS findings in patients with AKI.”

**[2] Reviewer #2 comments:**

Thank you for the response. I still have a technical question. Physiologically and anatomically it is well known that the blood flow and vessels of medulla are much less than that of cortex. It feels strange the blood flow of medulla looks similar to cortex in Figure 3. But it could be limitations for the quantitative CEUS as the authors discussed.

We thank the reviewer for this informative comment and agree with you. Unlike medulla, which normally receives less flow than cortex, we speculate that a medullary blood congestion and consequent slowing of the blood flow occurred in our patients with AKI. This medullary congestion is a hallmark of ischemic AKI in both experimental models (Kidney Int 1990; 37: 1240-1247, Kidney Int 1990; 38: 432-439, Kidney Int 1991; 40: 625-631, Lab Invest 1991; 65: 566-576) and in specimens obtained from biopsy or at autopsy (Kidney Int 1984; 26: 283-293). Currently, there is no CEUS study for medullary perfusion in humans with AKI. A recent animal study showed that medullary PI and AUC were about one-third of that of cortex in healthy dogs, on the other hand, medullary PI and AUC were almost the same as cortex values in dogs with AKI (J Small Anim Pract. 2019 Aug;60(8):471-476). The increase in medullary PI and AUC seen in dogs with AKI, which suggests increased medullary congestion, is similar to our results. However, our study had patients with mild AKI, whereas few papers on human CEUS had severe illness. Further studies with a larger sample size are needed for validation.

We added the discussion about the medullary CEUS findings as above.
